# Application of Deep and Machine Learning Using Image Analysis to Detect Fungal Contamination of Rapeseed

**DOI:** 10.3390/s20247305

**Published:** 2020-12-19

**Authors:** Krzysztof Przybył, Jolanta Wawrzyniak, Krzysztof Koszela, Franciszek Adamski, Marzena Gawrysiak-Witulska

**Affiliations:** 1 Food Sciences and Nutrition, Department of Food Technology of Plant Origin, Poznan University of Life Sciences, Wojska Polskiego 31, 60-624 Poznan, Poland or kprzybyl@up.poznan.pl (K.P.); jolanta.wawrzyniak@up.poznan.pl (J.W.); adams.franciszek@wp.pl (F.A.); marzena.gawrysiak-witulska@up.poznan.pl (M.G.-W.); 2 Department of Biosystems Engineering, Poznan University of Life Sciences, Wojska Polskiego 50, 60-625 Poznan, Poland

**Keywords:** rapeseed storage, mould, image analysis, convolutional neural networks, machine learning

## Abstract

This paper endeavors to evaluate rapeseed samples obtained in the process of storage experiments with different humidity (12% and 16% seed moisture content) and temperature conditions (25 and 30 °C). The samples were characterized by different levels of contamination with filamentous fungi. In order to acquire graphic data, the analysis of the morphological structure of rapeseeds was carried out with the use of microscopy. The acquired database was prepared in order to build up training, validation, and test sets. The process of generating a neural model was based on Convolutional Neural Networks (CNN), Multi-Layer Perceptron Networks (MLPN), and Radial Basis Function Networks (RBFN). The classifiers that were compared were devised on the basis of the environments Tensorflow (deep learning) and Statistica (machine learning). As a result, it was possible to achieve the lowest classification error of 14% for the test set, 18% classification error for MLPN, and 21% classification error for RBFN, in the process of recognizing mold in rapeseed with the use of CNN.

## 1. Introduction

Recently global demand for functional food is still growing among producers and consumers. Societies are looking for food, which has a substantial influence on improving their health condition, well-being, and reduces the possible risk of developing diseases. Functional food is fundamentally based on a diversified amount of bioactive compounds, among other things such as fiber, vitamins, and polyphenols, which have an influence on health. Having in mind the customer’s nutritional and dietary awareness and the need to follow a well-balanced diet, endeavors are made to look for products that will support the human organism and protect against health complaints and health condition. Rape, as one the plants belonging to the kind of oleaginous plants, is characterized by a high concentration of bioactive compounds such as tocopherols, phytosterols, beta-carotene, and polyphenol compounds [1,2,3,4]. Research that is currently being done shows that the use of rapeseed varieties that are free from harmful erucic acid allows for the obtaining of oil of better nutritional value [2,5]. It is also worth mentioning that winter rape as a cultivated oleaginous plant, is not only a source of plant oil for food industry and consumption but it is also cultivated all around the world as a raw material for feed, biodiesel [6,7,8,9,10] and biolubricant production [11]. Within the last several dozens of years (1994–2017), rape production has been continually growing and now amounts to 72.24 million tons (FAO 2018), and 17.81 million tons of rape is produced in Europe. According to statistical data from 2018, Poland is one of the ten countries in the world that has an influence on rape production and amounts to 2% of the world’s production (FAO 2018). On account of production scale and nutritional values of rapeseed oil, it also seems justified to place this oil in the food pyramid. Nevertheless, research shows that the quality of rapeseed oil is heavily dependent on the storage method, cultivar selection, and pre-harvest conditions [3]. Improper selection of storage conditions, i.e., incorrect temperature and seed moisture content higher than 7%, which is recommended in long-term storage of rape seeds [12,13,14] can lead to mold fungal growth [15,16,17] among other things, xerophilous kinds [18]. Therefore, it seems justified to search for modern, noninvasive methods that support the evaluation of the quality of the conditions of rape and eliminate contaminations including mold at an early stage of storage.

Currently, one of the most modern techniques of image processing [19,20] and image classification [21,22] in decision-making processes is the use of artificial intelligence methods [23,24]. In recent years, artificial neural networks have become so popular and effective that they started to be used in various problematic areas, among other things, in the optimization of food processes [25]. On account of the complexity of neural network processes supporting, among other things, visualization techniques [26], a proper formulation of decision-making problems is required, hence, it is possible to solve given problems. For the last couple years, Artificial Neural Networks (ANN) have been supported by Convolutional Neural Networks (CNN) [27,28,29]. The difference depends on the interpretation of network of input variables, in this case, images. The traditional technique of learning with ANN takes place by selecting, among other things, adequate parameters (descriptors) indicated in an image with previously carried out processing functions, filtration, edge detection, and texture, and with the use of learning algorithms such as the fastest neighbor method, Kohonen’s method, and the K-average method. Image pixels [30,31] are exclusively responsible for the parameter determining the input variables in CNN and the learning process of those networks requires the processing of more than two hidden layers (the so-called multi-layer learning directly from the input to output layer). In view of the above, in CNN networks it is not required to isolate the given functions from images before the learning process, as it takes place with machine learning [21]. In the literature, it is now observed that CNN networks substantially increase classification effectiveness in comparison with traditional methods of machine learning [32]. In view of the above, the research made a comparison of the effectiveness of detecting rapeseed affected by mold between Multi-Layer Perceptron (MLP), Radial Basis Function (RBF), and CNN networks.

The utilitarian goal of this research was to devise and compare neural models capable of fast and non-invasive identification of filamentous fungi affecting rapeseed stored in different humidity and temperature conditions. The model data constituted images of the morphological structure of rapeseed affected by mold to different degrees, obtained with a microscopic technique.

## 2. Materials and Methods

### 2.1. Preparation of Samples

The research material was rapeseed cv. PR46W20 with initial moisture content of 6.15 ± 0.21% harvested at The Experimental Agricultural Facility in Swadzim in 2019, belonging to Poznań University of Life Sciences. In order to obtain samples of rapeseed with different degrees of mold contamination, the selected seeds were subject to storage experiments. Prior to carrying out the experiment, the seeds were brought to the assumed storage conditions, i.e., 12 and 16% seed moisture content on a wet basis (w.b.) and 25 and 30 °C, according to the procedure described by Wawrzyniak et al. (2018) [17]. In order to do this, 4 kg samples of rapeseed were mixed intensively and sprayed with the proper mass of distilled water. The amount of water used to moisturize the seeds was determined on the basis of mass balance. The samples of seeds after moisturizing were put in polyethylene bags and then conditioned for 24 h at a temperature of 5 °C. Next, the moisturized rape seeds were stored in stable humidity and temperature conditions in the environmental chamber described earlier by Wawrzyniak et al. (2013) for the period of 30 days [33]. The constant moisture content in seeds was obtained by maintaining relative air humidity in spaces between seeds at a constant level with the use of saturated salt solutions (KCl, KBr, and BaCl_2_) present in the hydrogastric devices of the chamber. Relative air humidity balance corresponding to the requested moisture content of the seeds in both temperatures was determined with Halsey’s equation [34]. During storage, the temperature in the seed mass was monitored with thermocouples Cu-Konstantan, and relative humidity in spaces between seeds was monitored with humidity probes with capacitive sensors. Besides, during storage, the moisture content of the seed was monitored with an electronic moisture analyzer (Sartorius MA30, Germany). At the same time, at six-day intervals, samples were taken for further research aimed at the detection of seed contamination with filamentous fungi.

### 2.2. Image Preparation by Microscope

**Figure 1:**
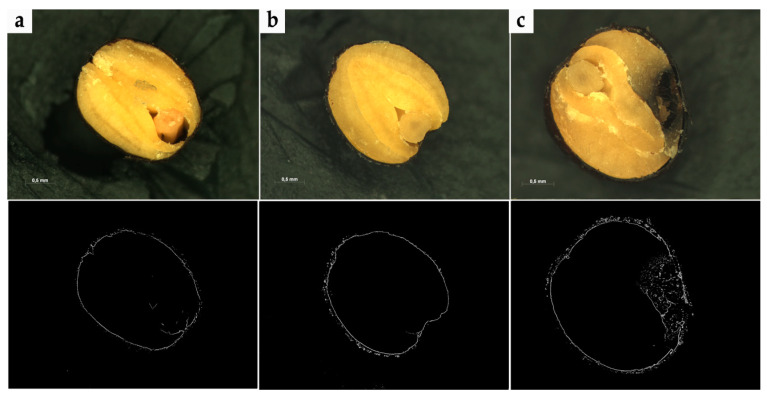
Examples of images that present rapeseed trials: (**a**)—rapeseed free of contamination with initial moisture content of about 6%, (**b**)—contaminated rape seeds stored in conditions characterized by 12% seed moisture content and at a temperature of 25 °C, (**c**)—contaminated rapeseed stored in conditions characterized by 12% seed moisture content and at a temperature of 30 °C.

**Figure 2:**
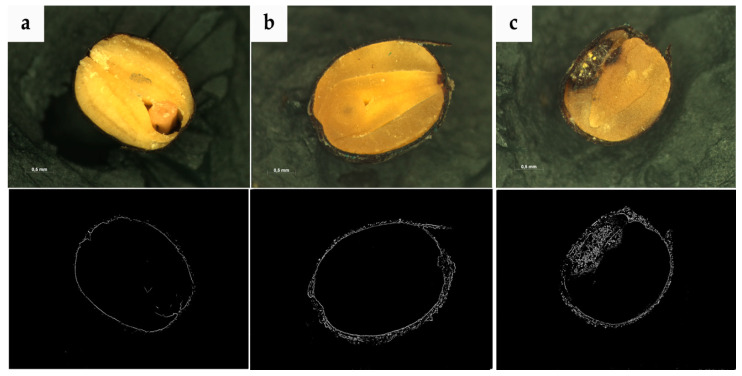
Examples of images that represent rapeseed trials: (**a**)—rapeseed free of contamination with an initial moisture content of about 6%, (**b**)—contaminated rape seeds stored in conditions characterized by 16% seed moisture content and at a temperature of 25 °C, (**c**)—contaminated seed stored in conditions characterized by 16% seed moisture content and at a temperature of 30 °C.

In order to acquire graphic data, isolated rapeseed with different degrees of filamentous fungi contamination were cut with a microtome. Next, the morphological structure of the isolated seeds was observed with a stereoscopy microscope. During the research, a series of digital images was used for the rapeseed samples. Examples of the trials are presented in Figure 1 and Figure 2. To acquire microscope images, an AxioCam MRc5 camera was used, which is characterized by high effectiveness when working with difficult specimens, among other things, rapeseed (seed size about 1–2 mm). A dynamic range of 1:1300 with 12-bit digitization ensuing loss-free image dynamics for optimal capture of various color intensities. Prior to acquiring image acquisition, calibration of the AxioCam MRc5 was also carried out, which plays a crucial role especially in achieving repetition with images from rapeseed trials. As a result, 520 images were acquired with a resolution of 2572 × 1936, 32-bit color depth, saved in.TIFF format. The research was carried out in The Electron and Confocal Microscopy Laboratory at the Adam Mickiewicz University in Poznań.

### 2.3. Image Processing by Software

**Figure 3:**
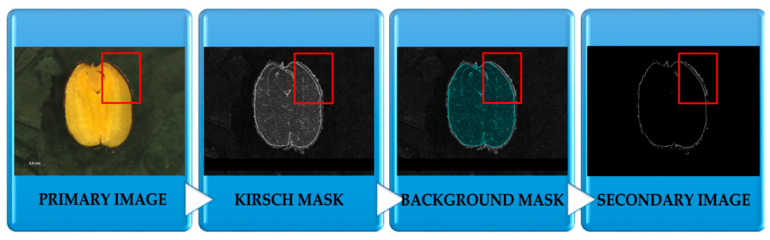
Scheme of secondary image acquisition using image analysis.

Another stage of the research required the preparation of ANN for the learning process, in this case, samples of rapeseed in the form of numerical data were used. In order to this, the MatLab environment and original software “PID system” were used. The first step required adaptation of the rapeseed sample in such a way to determine its features, i.e., the important standard of a digital image. Figure 3 shows stages of creating a standard from an image in order to isolate only fungi contamination in the rapeseed sample. Using the “PID system” in image processing and analysis in order to create the original image, which was acquired with digital microscopy, a filtration was carried out based on the maximum gradient with a Kirsch mask [35]. The Kirsch compass mask is used to attribute maximum magnitude to the central element with filtration of eight masks rotated to each other at 45 degrees [36]. In another stage, the cutting of the background inside the rapeseed sample was carried out. In order to do this, it was necessary to prepare the original script in MatLab supporting this way of cutting out image fragments. Secondary images were extracted to descriptors of image texture thanks to the use of the original software “PID system” with the module of batch image processing. The “PID system” allows for the extraction of image descriptors and the use of various operations on an image, including segmentation, filtration, and making masks (for example Laws’ masks) [19,37]. It needs to be added that the Gray-Level Co-occurrence Matrices (GLCM) matrix algorithm was used in the research in order to isolate descriptors of secondary images [19,38,39]. The aim was to achieve a better visual effect of substantial details for secondary images that were created. Rapeseed including pathogens, which are graphic objects, are very difficult to visualize because of their tiny size. The application of this technique allowed for fast, non-invasive isolation of details from an image on the basis of pixels with determined values appearing in an image. The “PID system” software together with the MatLab environment allowed for the creation of a learning set (file with cry extension) including 520 learning cases received from secondary images. Each case included 18 selected descriptors of image texture [38].

### 2.4. The Structure of the Neural Network

**Figure 4:**
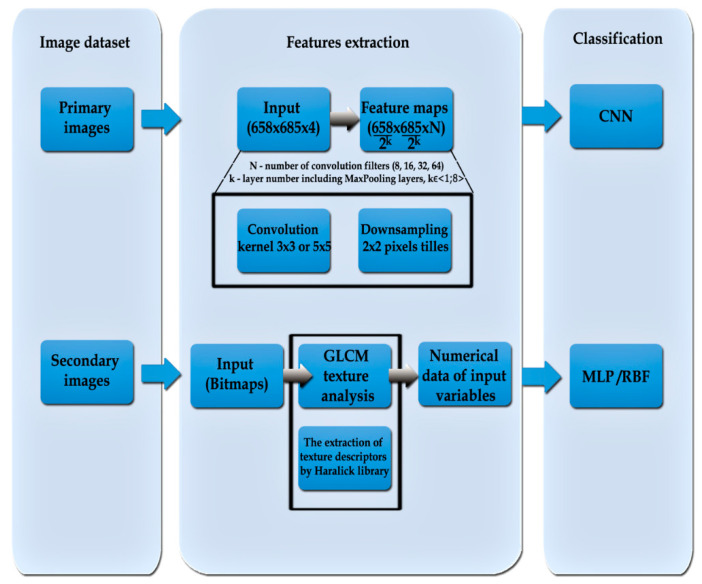
Algorithm schema of processing an image in the process of creating neural networks including deep and machine learning. In the case of CNN, the network model responded both to the extraction of features and to classification.

Within the scope of the research, networks were prepared on the basis of data characteristics for a given network type: MLP/RBF and CNN. The first method of designing a network was based on preparing a learning set Z1 in order to determine numerical data of texture from a secondary image. The process of acquiring data was carried out by image processing and analysis with the “PID system” and MatLab. As a result of the above procedure, 18 representative features of an image were obtained (variables such as angular second moment, contrast, correlation, sum of square variance, inverse difference moment pairs of pixels, sum of average pairs of pixels, sum of variance, entropy, difference variance, difference entropy, and info. The measure of correlation one and two, cluster prominence, cluster shade, dissimilarity, homogeneity, maximum probability, and inverse difference normalized) on the basis of GLCM matrix for each learning (secondary) [23,38,40,41,42,43]. The second method of designing a network was based on determining descriptors (secondary images) together with information determining fungi contamination of rapeseed, which were used to build up convolutional neural networks as input variables for learning set Z2. In order to improve the effectiveness of recognizing the primary image on the first stage, a rotation and image cutting with Python coding was carried out as well as the modification of images (moving and zooming, each time with random values) from the learning set individually for each sample before each learning session. Thanks to these operations, the model that was trained received slightly different images created from the same primary samples belonging to the learning set, which each learning session. After building learning set Z2, image convolution was used, from which a map of features with determined shape (width, height, and channels) was acquired from the microscopic image (primary image) with selected resolution. Each image pixel was assigned to model value “RGBA”. The structure of the devised CNN network included (Figure 4):

- Input layer, i.e., 685 × 685 × 4 bitmap, in which 32-bit primary images in RGBA format were included;
- Convolution layers for each loaded image, 8, 16, 32, or 64 filters were used. The kernel size in the first two convolution layers was 5 × 5, and 3 × 3 in the next two. Parameter “strides” determined the dimension of the convolution step (the distance of the convolution window that is moved) expressed as (width, height) was set as value (2, 2) [44,45];
- The pooling layer was used to reduce information included in previously acquired images. Operation MaxPooling allowed for the reduction in the resolution of bitmaps (learning case). For each map of features, 2 × 2-pixel areas were singled out. For each region, the maximum pool algorithm was used, i.e., the resultant pixel with the highest value was selected, and other pixels were omitted. Each operation resulted in image reduction in the outermost areas of this map. The “padding” parameter was set as “same”. It means the outermost areas of the matrix field that was processed were added after each MaxPooling operation in order to avoid excess data loss. As a result of this, the data size of the operation was the same as the size of input data. Convolution depth was doubled for each two-fold width and height reduction of the analyzed image. It minimized the problem of fitting networks to training sets via gradual resolution reduction of the processed image during tensor operations. Reduction of resolution on the stage of initial data processing could lead to the loss of some data and would affect the prediction abilities of the created network;
- A thick (fully connected) layer including 288 neurons with activation function “relu”, and one neuron with activation function “sigmoid”. The function of activating a rectified linear unit, causes it to return the standard activation ReLu with default values within the range: max (y, 0), for which basic maximum equals 0 and value “y” is treated as an input tensor. On the other hand, for low values (<=5) sigmoid returns a value close to zero, for high values (>5) the result of the function is close to one.

## 3. Results and Discussion

### 3.1. Results of Machine Learning

**Figure 5:**
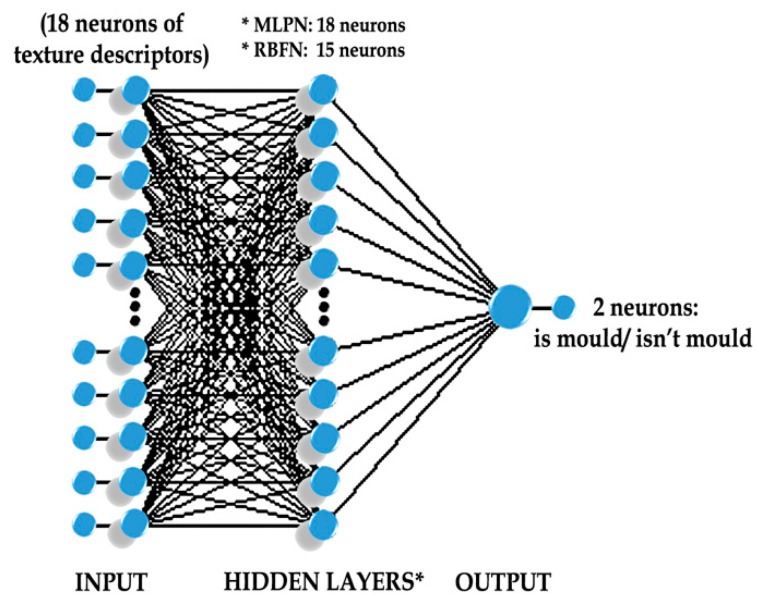
Structure of selected MLP neural networks with 18 neurons in the hidden layer (MLPN) and RFB neural networks with 15 neurons in the hidden layer (RBFN).

ANN simulations were carried out with deep and shallow learning. In the process of generating networks with the method of machine learning, Statistica software was used. As a result, RBF and MLP typologies were tested. It resulted that MLP networks were characterized by higher classification accuracy than RBF networks (Table 1). Among MLP-type networks, the one which received the lowest RMSE rate was selected. Table 1 presents the results of the machine learning process and determined, selected input variables (18 descriptors of image texture). An adequate MLP network was created on the basis of 18 input variables, 13 neurons in the hidden layer, and 2 neurons in the output layer (Figure 5). Effectiveness of an MLP 18:18-13-2:1 network was reached on the level: 0.89, RMSE: 0.17, and MSE: 0.03 on account of the rate of proper classification (accuracy). During the process of machine learning, BP algorithms were used, i.e., the function of learning with the backpropagation algorithm and the function of conjugate gradient CG. In the case of the RBF network, worse classification effectiveness was observed than in MLP and CNN networks. For comparison, the RBF 18:18-15-2:1 network structure was characterized by the same input variables (texture parameters) such as in the case of the MLP 18:18-13-2:1 network, 15 neurons in the hidden layer, 2 neurons in the output layer (Figure 5). RMS error in RBF 18:18-15-2:1 was 0.21 and MSE: 0.04. During the process of learning the RBF network, K-Means (KM), K-Nearest Neighbor (KN), and Pseudoinversion (PI) algorithms were used. The main aim in the process of the learning network was to reach the lowest RMSE error. In analyzing the level of difficulty in quick and effective recognition of pathogen (on account of different stages of its development) in rapeseed, different variants of a selection of input variables, i.e., descriptors of image texture, were taken into consideration. It is worth noticing that the GLCM matrix includes many more Haralick’s descriptors [23,38]. Unfortunately, using all texture discriminants caused the networks to overlearn. It concerned both RBF and MLP typology.

### 3.2. Results of Deep Learning

**Figure 6:**
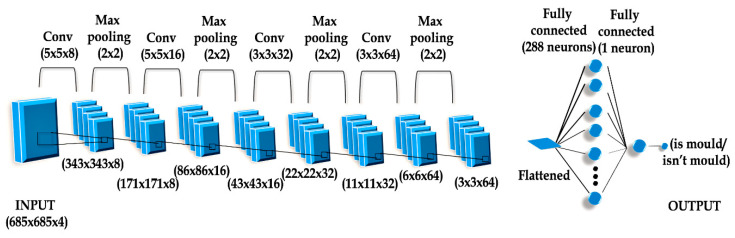
Structure of adequate Convolutional Neural Networks.

Table 1 presents a comparison of network results with deep learning. The process of network simulation was carried out in the Anaconda environment (https://www.anaconda.com/), framework Tensorflow [45] and Keras library supporting image classification [44]. The optimal CNN network, which was characterized by the highest effectiveness in detecting a pathogen in rapeseed consisted of an input layer, three hidden convolution layers, one hidden layer of thickly connected units, and one neuron in the output layer determining a two-state variable informing about the presence or lack of presence of the pathogen (Figure 6). Each learning case determined the bitmap of a primary image, i.e., as it was mentioned in methods, it is a 685 × 685 resolution image with 32-bit color depth acquired with a stereoscope. The four first layers constitute alternant laid hidden layers Conv2D with Max Pooling. After convolution series, the multidimensional tensor variable was reduced to a univariate variable and transferred to another layer of thickly connected neurons [45]. In the last layer, there was only one neuron acting as an adder returning a single value from the range (0, 1). An adaptive algorithm, “Adam”, was used as an optimizer. Function “relu” was used as the activation function in the first layers and in the hidden layers. On account of the coefficient of accurate qualifications (accuracy) it was possible to achieve the effectiveness of a CNN network on the level of: 0.89, the RMS error value was: 0.24, and MSE value was: 0.06.

On the basis of the literature and research experience, it is possible to confirm the effectiveness of pathogen identification on rapeseed with the use of deep learning. Despite the fact that the analyzed trials of rapeseed were affected by fungi to a lesser or greater degree, the application of deep and machine learning allowed for the achievement of satisfying results with a high degree of accuracy. Very good results of the identification of fungi contamination in rapeseed were achieved with machine learning but the MLP network was characterized by a higher testing value (0.90) than in the case of an RBF network whose testing value was (0.85). Finally, the best effects of learning for the learning set on the level of 0.97 was achieved with the simulation of a CNN network. The optimal CNN network was characterized by the lowest testing error. The reason for weaker results with machine learning could be the difficult size (a diameter of about 1–2 mm) and morphological structure of rapeseed. Nevertheless, further research is being done in order to optimize this process. The application of modern techniques, including neural networks, will allow for the optimization of the process of contamination detection, including fungal contamination of rapeseed in the early stage of storage. It is worth pointing out that in the face of shrinking cultivation areas, it seems justified to look for methods facilitating an effective crop of oleaginous plants. In the future, there is an intention to implement such models, for example, in expert systems, which would allow, among other things, a non-invasive massive scale evaluation of the quality condition of rapeseed.

## 4. Conclusions

On the basis of the research carried out, it was found that classification error of fungi contamination in rapeseed with the use of deep and machine learning for the devised networks fluctuated between 14 and 21% in relation to the testing set.

It was observed that in the case of deep learning with a CNN network, a similar rate of classification accuracy on the level of 0.89 was achieved, as in the case of machine learning with an MLP network. In case of the RBF network, the rate of classification accuracy was 0.85.

It turned out that the topologies of CNN and MLP networks were a more accurate tool for identifying the stage of fungi condition of rapeseed than the typology of RBF network.

It can be observed that the reduction in the number of input variables in the MLP network had an influence on improving the effectiveness of recognizing the quality condition of rapeseed.

## Figures and Tables

**Table 1:** Results of network learning.

Name Learning Set	Z1	Z1	Z2
Model ANN	MLP	RBF	CNN
Training error	0.19	0.22	0.29
Validation error	0.15	0.19	0.30
Testing error	0.18	0.21	0.14
Quality of learning	0.88	0.87	0.87
Quality of validation	0.90	0.85	0.84
Quality of testing	0.90	0.85	0.97
Learning cases	520	520	320
Training algorithm	BP50, CG315b	KM, KN, PI	Adam
